# Psychological Well-Being From Sports Injuries in Adolescence: A Narrative Review

**DOI:** 10.7759/cureus.64018

**Published:** 2024-07-07

**Authors:** Lauren Jeong, Dan Li

**Affiliations:** 1 Psychology, Farmington High School, Farmington, USA; 2 Medicine, Yale University, New Haven, USA

**Keywords:** athlete identity, youth sports, psychology, sports injuries, adolescent

## Abstract

The objective of this paper is to review how the mental and psychological well-being of the adolescent athlete population may be impacted by sports injuries, specifically the development of their personal identities.

To answer this question, we conducted a narrative review using keywords such as "adolescence," "psychological," "injury," and "sport" in the PubMed database. When conducting the research, we included sources from the past 15 years in order to gain a more present and accurate analysis of our question, and no countries were excluded from our population.

Through our research, we identified risk factors that contribute to the rising psychological stress on adolescents. The return to sport was also found to be primarily affected by implied psychological illnesses such as self-confidence and identity, along with parental and coach guidance through the rehabilitation process. Despite the lack of research, scientists work to pursue additional psychological interventions to ensure the emotional well-being of adolescent athletes.

Through this review, we aim to inform athletes, coaches, parents, and pediatricians about psychological issues that they may face as they continue to pursue their respective roles in sports. This study also paves a path for future research concerning potential interventions to prevent such psychological issues and ensure both physical and mental health for young athletes.

## Introduction and background

Introduction

The past several decades have seen the exponential growth of sports as an expected extracurricular for younger children [1]. Similarly, implications of growing mental health problems have emerged globally alongside the advancement of widespread technology and the recognition of the importance of psychological well-being in the adolescent population [1]. As sports continue to develop into a significant aspect of social communication and involvement in a community, issues surrounding injury arise simultaneously. Disturbances in physical and mental growth during this crucial and sensitive period for adolescents raise the question of neuropsychological development as well [1].

Previous investigations have identified certain physiological aspects of injuries related to sports [2]. However, these existing accounts have failed to address the harmful psychological impacts on athletes after such setbacks. As athletes reach puberty and sports rapidly grow competitive, high-pressure settings can cause increased psychological demands. This is compounded as each athlete experiences various development and skill acquisitions over time, adding to the rising expectations of being great in the sport [3]. Much uncertainty still exists about the nurture aspect of the nature versus nurture argument concerning the maturity of athletes following injury. A study dedicated to researching the self-development of athletes following adversity will seek to better the future of research for young athletes and their support systems.

This research aims to highlight the importance of mental health in the world of sports and examine the relationship between an overuse of sports injuries and psychological well-being, especially the impact on identity and self-worth. We hypothesize that there are several psychological consequences related to overtraining after sustaining sports injuries. Motivation and persistent diligence are a part of the identity of many athletes, so we suspect that overtraining is a natural response to their rigorous work ethic. However, failure to achieve their desired results in their respective sports may cause additional psychological complications such as anxiety or depression, and long-term effects may cause disorders such as post-traumatic stress disorder (PTSD). The purpose of this study is to guide future areas of research and explore a new direction to analyze and support the common issue of mental health issues in young athletes.

Our narrative review has been divided into three major parts. We will first introduce the methodology of our studies, which includes our research question and subsequent overview; inclusion criteria regarding the participants; concept, context, and source of evidence; the search strategy we used through PubMed; our screening protocol; and finally our flowchart and data extraction policies. The next section of this paper presents the findings of our research. This part will highlight the data that we found through our search strategy, with a focus on the description/type of problem, risk factors, relationships, and interventions. In the end, we will discuss more in-depth about why our findings are significant to the future of research. Throughout this paper, the terms "training" and "athletic" will be used to refer to sports, "mental" and "psychological" will be used similarly to express nonphysical topics, and the terms "youth," "adolescents," and "young athletes" will likely be used to address the adolescent age of approximately 10-18, when the majority of the population goes through puberty.

Methodology

Research Question

How does injury from sports in adolescents affect their psychological well-being and development of identity?

Subquestions

Derived from our focus question, we formed additional questions to zoom in on specific aspects of our research. These questions include the following: "What has been found on the frequency of injuries in young athletes?" "What effect does increasing age have on the psychological demands of young athletes?" "How often do young athletes return to their sport following an injury?" "How does the critical period of adolescence impact the development of self-identity?" "What are the effects of specific injuries derived from overuse on confidence in young athletes?"

Inclusion Criteria

In our systematic review, we focused on a specific population and controlled our research to the specifics of the psychological implications of sports injuries in adolescents. Explicitly, particular standards were evaluated to write our paper. In terms of types of participants, the target participants in this review consist of athletes of various sports who have sustained injuries, specifically in their adolescent years. The age group studied was between 10 and 18, when most of the youth population go through puberty. The concepts we focused on were a broad topic of an overview of the injuries in youth athletes in adolescence, zooming into natural psychological development in young athletes versus young nonathletes, and types of psychological mechanisms that can cause an overuse of sports injury in the youth population. The context of this review includes all countries, and in this review, the primary database used was PubMed. We used the data extraction process in order to identify pertinent sources to the topic researched.

PubMed Search Strategy

To gather our research, we used PubMed, in which we entered several search terms to produce relevant sources. Ultimately, the search strategy we used was as follows: (("adolescences"[All Fields] OR "adolescency"[All Fields] OR "adolescent"[MeSH Terms] OR "adolescent"[All Fields] OR "adolescence"[All Fields] OR "adolescents"[All Fields] OR "adolescent s"[All Fields]) AND ("injurie"[All Fields] OR "injuried"[All Fields] OR "injuries"[MeSH Subheading] OR "injuries"[All Fields] OR "wounds and injuries"[MeSH Terms] OR ("wounds"[All Fields] AND "injuries"[All Fields]) OR "wounds and injuries"[All Fields] OR "injurious"[All Fields] OR "injury s"[All Fields] OR "injuryed"[All Fields] OR "injurys"[All Fields] OR "injury"[All Fields]) AND ("sport s"[All Fields] OR "sports"[MeSH Terms] OR "sports"[All Fields] OR "sport"[All Fields] OR "sporting"[All Fields] OR "Return to Sport"[Mesh] OR "Youth Sports"[Mesh]) AND ("psychologie"[All Fields] OR "psychologies"[All Fields] OR "psychology"[MeSH Subheading] OR "psychology"[All Fields] OR "psychology"[MeSH Terms] OR "psychology s"[All Fields])) AND ((systematicreview[Filter]) AND (2009:2024[pdat])).

Screening Protocol

In our systematic review, we conducted two rounds of screening. The first round screened the title and abstract, and the second round screened the full text of the manuscript retrieved by our search strategy.

Flow Chart/Data Extraction

We used the Preferred Reporting Items for Systematic Reviews and Meta-Analyses (PRISMA) flow chart in order to illustrate the numerical outputs from scoping reviews and the inclusion decision process. The flow chart clearly outlines the process of finding studies, removing duplicates, selecting relevant research, retrieving the full text from the library, and presenting the final analysis.

We also developed a charting template (Table 1) regarding the characteristics and key points of the articles we included in our systematic review in order to facilitate data extraction.

**Table 1:** Flow Chart and Data Extraction for Papers Reviewed PROs, patient-reported outcomes; PTSD, post-traumatic stress disorder; OCD, obsessive-compulsive disorder; RSI, Return to Sport after Injury; I-PRRS, Injury-Psychological Readiness to Return to Sport; GPA, grade point average

Paper	Outcome Measured	Main Findings	Participant Age	Population Characteristics	Intervention	Limitations	Measured Variables	Study Design	Country	Year of Publication	First Author	Sample Size	Type of Injury	Psychological Variable
Physical Activity and Sports-Real Health Benefits: A Review with Insight into the Public Health of Sweden	Health-related physiological effects of aerobic and muscle-strengthening physical activity	There is strong scientific evidence supporting an association between physical exercise/training and good physical and mental health	-	-	-	-	-	-	-	2019	Christer Malm [1]	-	-	Depression, psychological stress, motivation, and psychological abuse
Self-Reported Symptoms of Depression and Anxiety After ACL Injury: A Systematic Review	Symptoms of depression and anxiety assessed	Symptoms of depression and anxiety are more severe in early stages after anterior cruciate ligament (ACL) reconstruction, with depression being more common among professional athletes	16-65	Male, 60%; female, 40%	-	Small number of studies/patients included, self-reported symptoms of depression and anxiety, and not clinical diagnosis	Presence and severity of self-reported symptoms of depression or anxiety given on psychometric-tested PROs	Systematic review	Gothenburg, Sweden	2022	Ramana Piussi [4]	682	ACL injury	Diagnosed conditions (depression and anxiety)
Mental Health in the Specialized Athlete	Psychological consequences of early sports specialization including anxiety, depression, maladaptive perfectionistic traits, overtraining, clinical eating disorders, impact on performance, physical health, and overall well-being	Early sports specialization is associated with increased risk for injury and burnout, leading to potential mental health issues such as depression, anxiety, and maladaptive perfectionistic traits	12-18	Athletes specializing in a single sport at an early age, elite athletes delaying specialization until mid-late adolescence, and youth athletes with varying levels of competitive identity and anxiety, fear of rejection, and academic pressures	Working within the context of early specialization to determine how best to prevent injury and burnout and optimize the physical and psychological development of young athletes and the implementation of mental health literacy programs for athletes, parents, coaches, and youth sport organizations	Need for further work and investigations to better inform sport-specific recommendations and explore the efficacy of interventions	Early sports specialization, risk for injury and burnout, mental health implications, likelihood of participating at college/professional level, and the risk of depression in athletes	Systematic review	-	2023	Mary M. Daley [5]	-	Injuries in specialized athletes	Burnout, anxiety, depression, shame, perfectionism, and disordered eating
Psychosocial Aspects of Sport-Related Concussion in Youth	Psychological and social impact of sports-related concussions (SRC) on individuals, families, and communities	SRC has significant impacts on individuals, and media portrayal influences recovery and participation and gaps in research on consequences and recovery	0-19	Increasing trend in concussion in youth athletics over the past several decades, decline in participation in high school sports in recent years, and high level of concern from parents about children being exposed to SRC	Gradual return to preinjury activities, more conservative rehabilitation strategies, and collaboration across disciplines involving coaches, trainers, and parents	Mixed data and gaps in medical research	-	-	United States	2021	Aaron S. Jeckell [6]	-	Concussions	Physical integrity, fear, and trauma
Sports Specialization in Young Athletes: Evidence-Based Recommendations	Rates of sports specialization in young athletes, risks of single-sport intense training, and injury incidence in athletes competing at different levels	Risks of single-sport intense training include adverse psychological stress and premature withdrawal from competitive sport	8-18	Children and adolescents, with increasing trends in sports specializations among high school students	-	Limited data from studies with small sample sizes and retrospective design, reliance on expert opinion due to limited data, and inconsistent demonstration of essentiality of early intense training for attaining elite level in all sports	Rates of sports specialization in young athletes, presenting for sports physicals	-	United States	-	Neeru Jayanthi [7]	243	-	Burnout and fatigue
Mental Health in the Youth Athlete	Depression, anxiety, PTSD, emotional lability, cognitive difficulties, and behavior responses	Current trends in youth sport participation and mental health of adolescent athletes after school closures that led to increased depression and anxiety, lower physical activity levels, and decreased quality of life scores	5-21	-	-	-	Athlete Identity Measurement Scale (AIMS), Pediatric Symptom Checklist (PSC), and Strengths and Difficulties Questionnaire (SDQ)	-	United States	2023	Mary M. Daley [8]	-	Musculoskeletal injury, concussions, and injury overall	Athletic identity, depression, anxiety, burnout, perfectionism, fear, and PTSD
Psychosocial Impacts of Sports-related Injuries in Adolescent Athletes	-	Significant mental health benefits of sport participation for young athletes, the lack of research on the outcomes of youth athletes following injury, and need to understand the psychosocial impact of sports injuries in this population	12-21	Adolescents, both male and female, including those with self-reported recent injuries and navigating changes in independence, identity, and athletic development	Relaxation, mindfulness, imagery, goal setting, and stress management for six months following injury	Lack of available information regarding psychosocial impacts of injuries in adolescent athletes	-	-	United States	2021	Kristin Haraldsdottir [9]	-	ACL injury	Diagnosed conditions (depression, anxiety, and PTSD) and personal identity/self-esteem
Stronger Athlete Identity Is a Risk Factor for More Severe Depressive Symptoms After Musculoskeletal Injury in Pediatric Athletes: a Systematic Review	Association between musculoskeletal injury and poor mental health and functioning in pediatric athletes	Musculoskeletal injury is associated with worse mental health in pediatric athletes, stronger athlete identity is a risk factor for the development of depressive symptoms, and psychological interventions that reduce uncertainty and address fear may help mitigate these risks	14-21	Pediatric athletes with a significant role of sports in their lives and potential parent/guardian involvement in their sports activities	-	Focus on knee injuries, variety of sports studies, the lack of validation for psychological health measures in athletes or pediatric populations, and the absence of discussion on social determinants of health	Athletic identity, health-related quality of life, mood/depressive symptoms, anxiety, PTSD symptoms, fear avoidance, and physical health measures	-	-	2023	Anna L. Park [10]	-	Musculoskeletal injury, concussions, and injury overall	Identity, uncertainty, fear, anxiety, depression, PTSD, and OCD
Psychological Aspects of Adolescent Knee Injuries	Psychological impact of knee injuries on adolescents, development/exacerbation of underlying psychological disorders, symptoms of PTSD, rates of depression, and fear avoidance	Knee injuries in adolescents can lead to both physical and psychological disturbances	10-21	Adolescents participating in sports such as soccer and football and racially diverse population participating in competitive athletics in high school	Aggressive/frequent rehabilitation programs with focus on psychological aspects	Limited analysis of psychological aspects of injury and recovery in adolescents, narrative nature of review, reliance on existing literature for therapeutic recommendation, and need for further research	ACL-RSI score, Tampa Scale of Kinesiophobia (TSK) score, Athletic Coping Skills Inventory-28 score, fear avoidance, self-efficacy, and athletic identity	-	-	-	Aneesh G. Patankar [11]	-	Knee injury	PTSD, depression, athletic identity, and fear
Weightlifting for Children and Adolescents: A Narrative Review	-	Properly supervised resistance training programs can improve sport performance, reduce injury potential, and enhance health aspects in children and adolescents	7-22	Youth involved in weightlifting, both male and females	-	Ambiguity and controversy surrounding the psychological basis for improvements in motor performance throughout childhood and adolescence	Strength improvements, measures of cardiorespiratory fitness, hormonal responses and adaptation, menstrual cycle characteristics, and performance-related, physical, and physiological variables	-	-	2021	Kyle C. Pierce [12]	70	Injuries in weightlifting athletes	Self-esteem and overall psychological development
Youth sport: Friend or Foe?	Overall physical fitness, improved bone health, and protection against future morbidity	Sport participation in childhood and adolescence is associated with a wide range of physical, mental, and social benefits	10-18	-	-	Lack of longitudinal data on youth sport outcomes, need for emphasis on injury prevention and better injury management strategies, and requirement for a better understanding of the interacting effects of maturation, training load, and sport type	Physiological, biomechanical, and psychological growth during puberty; menstrual irregularity; and athletic identity	-	England	2019	Carly D. McKay [13]	1387	-	Athlete identity
Psychological and Social Components of Recovery Following Anterior Cruciate Ligament Reconstruction in Young Athletes: A Narrative Review	Psychological factors associated with recovery after ACL injury and subsequent reconstruction in young athletes	Psychological factors are correlated with the ability of young athletes to return to play after ACL reconstruction	-	Young athletes participating in organized sports, specifically female soccer players and male football players	Postoperative psychological rehabilitation	Not all relevant literature was considered for inclusion; the selection of articles was solely that of the authors = potential interpretation bias	Psychological readiness (ACL-RSI and I-PRRS scales), fear of reinjury (TSK-11), motivation, and psychological rehabilitation	-	United States	2021	Emil Stefan Vutescu [14]	-	ACL injury	Psychological readiness, fear of reinjury, and desire to return to sport
Special Considerations for Growing Dancers	Sleep duration and quality in young dancers, including the mean hours of sleep per night and percentage of dancers reporting less than eight hours of sleep per night	Sleep duration and quality are crucial for the health maintenance of young dancers; young dancers are at risk of mental health problems, eating disorders, and negative metabolic and musculoskeletal health effects	12-17	Young dancers	Training modifications during the adolescent growth spurt, the evaluation of dancer sleep duration and quality, cognitive behavioral therapy for dancers with perfectionism, and the screening and early intervention of dancers with low energy availability	-	Mean hours of sleep per night, energy availability, menstrual disturbances, and hormonal panels	-	Canada	-	Bridget J. Quinn [15]	-	Injuries in dancers	Perfectionism, burnout, eating disorders, stress, and anxiety
Depression, fear of re-injury and kinesiophobia resulted in worse pain, quality of life, function and level of return to sport in patients with shoulder instability: a systematic review	Depression, fear of reinjury, and kinesiophobia associated with pain, function, quality of life, and return to sport activity in people with shoulder instability	Psychological factors such as depression, fear of reinjury, and kinesiophobia are associated with worse pain, quality of life, function, and the level of return to sports in patients with shoulder instability	-	Predominantly males with shoulder instability, most younger than 40	-	Exploring psychological factors through a narrow lens, not equally exploring all psychological factors, limited generalizability of findings due to few results, and cautious evaluation of results	Depression, kinesiophobia, and fear of reinjury	Use of different tools from Joanna Briggs Institute (JBI) Critical Appraisal for assessing the risk of bias in different types of studies	-	2022	Fabrizio Brindisino [16]	270	Shoulder injuries	Depression, fear, and kinesiophobia
Academic Performance Following Sport-Related Concussions in Children and Adolescents: A Scoping Review	Number of school days missed, changes in baseline academic performance, types and amount of school accommodations utilized, and self-reported measures of executive dysfunction	Statistically significant implications on academic performance decline after SRC and the scarcity of available literature on direct consequences of SRC on student academic performance and quality of life	5-18	Children and adolescents who have experienced sport and recreation-related concussions, including various severity of traumatic brain injury and comparison with extremity injuries	Four-step graduated return to school protocol with specific time restrictions and accommodations for students who continue to have symptoms with cognitive exertion in school	Different in age ranges and the lack of understanding on the impact of multiple factors on the course of recovery following SRC, need for further research to better understand how to implement accommodations in the student's learning environment, and findings from a limited number of studies	GPA, Pediatric Quality of Life Inventory version 4.0, qualitative survey methods, numerical measures, and subjective survey methods	Cross-sectional studies, cohort studies, case series, prospective longitudinal study, and population-based retrospective before and after study	-	2020	Mekala Neelakantan [17]	14	Sports-related concussions	Academic performance
Psychologists' Role in Concussion Assessments for Children and Adolescents in Pediatric Practice	Concussion assessments for children and adolescents in pediatric practice	Role of psychologists/neuropsychologists in concussion assessments for children and adolescents in pediatric practice and ability to provide expanded assessment capabilities and assistance with the prevention, identification, and management of multiple illnesses beyond concussions	Children and adolescents	-	Psychological services	Lack of extensive research on the role of psychologists in pediatric concussion care, limited awareness, and the potential lack of representation of psychology trainees in nonuniversity healthcare settings	Concussion symptomology and cognitive assessments	-	United States	2020	Roger W. Apple [18]	-	Concussions	Diagnosed conditions

## Review

PRISMA

**Figure 1:**
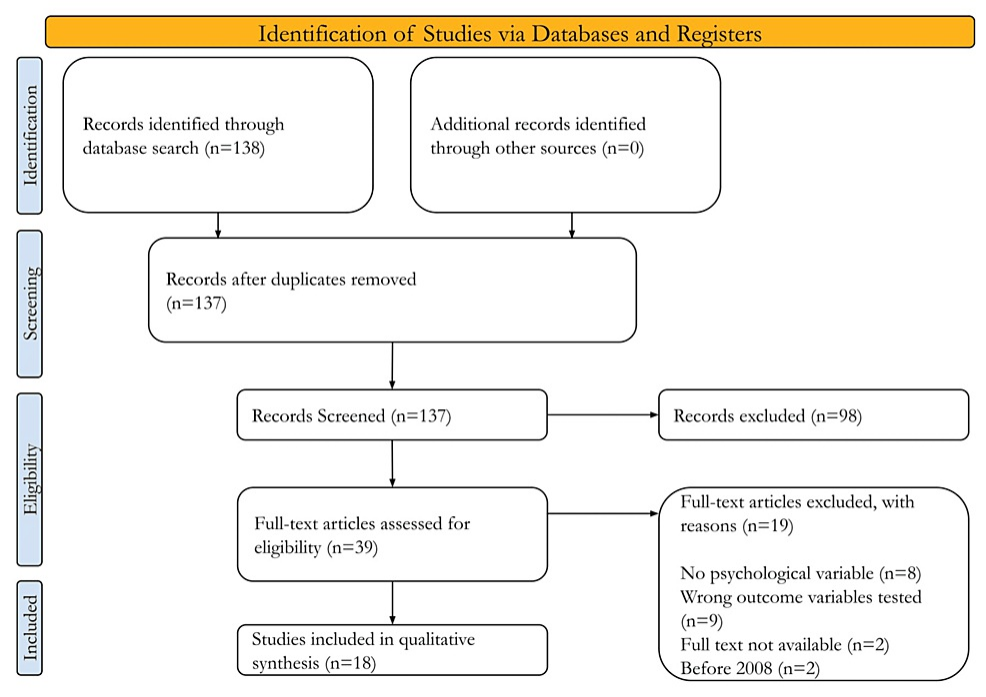
PRISMA Flow Diagram of Records Screened for Eligibility PRISMA: Preferred Reporting Items for Systematic Reviews and Meta-Analyses

To select the studies for data extraction, we began with 138 articles from our search strategy in the PubMed database. After removing one duplicate, we conducted our first round of screening based on titles and abstracts and excluded 98 articles, leaving us with 39 to be screened for full-text analysis. Finally, we were left with 18 articles to be studied for this narrative review after excluding 19 studies based on various factors such as a lack of outcome variables, limited access, and not fitting into our original search (Figure 1).

Paper characteristics

Ultimately, we narrowed down the papers to 16 studies based on different paper characteristics as shown in Table 1. The included studies were published between 2018 and 2023. These articles were conducted in two different continents: 14 in North America (the United States, 13; Canada, one) and two in Europe (England, one; Sweden, one) The studies included participants from age five to 65 but primarily focused on children and adolescents. Five studies focused primarily on depression and anxiety as psychological effects after injury, and 11 looked more broadly at mental health implications following sports injuries. Fifteen of the papers selected were reviews, with two specified as narrative reviews, one as a scoping review, and three as systematic reviews. There was also one evidence-based recommendation selected.

Description/type

Injuries

The effects of injury vary between the type of injury sustained. Of the papers chosen, some looked at broad spectrums of injuries, while others addressed a specific sport. Four of the papers studied looked at sports injuries, three focused on sports-related concussions, and four focused on musculoskeletal injuries, specifically anterior cruciate ligament (ACL) injuries. Meanwhile, two papers zoomed in on sports specialization, one looked at dancers specifically, one at weightlifting, and one at shoulder instabilities.

Many injuries have mental implications as a result of physical onset [4]. Factors such as early sports specialization can contribute to increasing rates of lasting negative mental impacts on athletes [5]. Participation in sports in childhood and adolescence is not without the potential for exposure to injury [6]. Injury incidence is significantly higher in athletes competing at an international level as compared to those competing at a recreational level [7]. The risk of injury depends heavily on the adolescent training ground and commitment to the sport. With such environmental pressures, early sports specialization has grown to be much more common, and factors such as age, competitive level, growth rate, and pubertal maturation stage only increase risks for both short-term and long-term negative impacts on mental health [7]. Mechanisms of injury derived from overuse and burnout can be a result of intense training, often leading to low self-esteem, an internal need to be perfect, a decreased locus of control, heightened anxiety, and a lack of confidence in one's abilities in relation to the excessive demands of social pressures [8]. In summary, the type and mechanism of injury often have great implications on the subsequent recovery process.

Return to Sport

Following an injury, the issue of returning to sport (RTS) with previous levels of play and mental stability arises. Adolescents often feel more pressure to return to play quickly, which only hinders the true physical and psychological readiness of athletes to return. One of the primary reasons for low return to play rates following injury was a difference in lifestyle following injury and the fear of reinjury [9]. The patients who return to their preinjury level of sport yet do not perform as well report similar reasons [4]. The increased likelihood to return to sport is more dependent on one's confidence in their ability, support at all levels, and active strategies to promote cognitive awareness of stressors with conscious efforts to reduce negative intrinsic feelings such as social isolation [10]. One interview study characterized the three main processes of RTS as driving reasons, the preparation of body and mind, and risk acceptance, all of which involve a connection between the physiological and psychological acknowledgment of one's capabilities and mental readiness to return [10]. On the other hand, the barriers that counter the drive to return include physical limitations, social factors, fear, and uncertainty about progress in rehabilitation [10]. It is essential that the athlete is completely ready to return to reduce the risk of reinjury and fully reach the previous level of play.

Short-Term Psychological Impact

Consequences of sports injuries may carry on until the physical injury is overcome or until all issues mentally and physically have been completely resolved. As Table 1 shows, some papers referred to psychological effects as diagnosable illnesses such as depression and anxiety, while others looked more abstractly in terms of athlete identity such as fear and perfectionism. It has been found that sport participation for adolescents is positively correlated with multiple positive indicators of physical and mental health. Adolescents are likely motivated to commit deeply to sports due to passion, a desire to foster unique skills derived from the sport, a drive to please and/or impress others, or the belief that it will lead to long-term athletic achievement [5]. Additionally, as seen in Table 1, the five main themes associated with the quality of life after injury are relationships, uncertainty/fear, mood, stress/pressure, and energy [10]. Premature RTS can exacerbate any injury and prolong recovery [6]. In the process of rehabilitation, the patients can develop negative psychological reactions, and when incorrectly addressed, they can negatively affect rehabilitation and treatment outcomes, continuing an endless loop that prevents the athlete from feeling confident to return to sport [4]. Psychologically, any injury or experience that leads to removal from one's sport can be extremely threatening to the athlete. Even a minor injury can cause athletes to question their integrity and identity as athletes, leading to distress and possible trauma. This trauma can also be influenced by a sense of responsibility to one's family and community [6]. Therefore, it is imperative that an athlete's mental well-being is monitored directly after the injury is sustained.

Long-Term Psychological Impact

Long-term consequences of an overuse injury are much more prominently based on mental hindrances to the quality of life and psychosocial health. Injuries in adolescence can interfere with psychosocial development during the important developmental stage in which athletes are finding their identities, which can lead to negative lifelong consequences [9]. This explains why children and adolescents are at a higher risk of long-term brain injury, even when the short-term symptoms of the trauma are resolved. Physically, the sudden secession of intense training may reduce blood flow to the brain, including the hippocampus, which is possibly related to the long-term decline in mental capacity [1]. Young athletes who have been pressured to perform to excessively high standards are likely to internalize athletic failures as feelings of shame, which can lead to unstable perfectionist traits, clinical eating disorders, or other harmful behaviors that will only result in further declines in performance and overall well-being [5]. These effects of injuries in adolescence can impair the quality of life even into adulthood, with an increased risk of anxiety, depression, and PTSD carrying into later life [9]. Even if the immediate risk seems to have passed, the long-term implications as the athlete grows into adulthood can cause further instability in the future and should be checked regularly.

Risk factors

The risk factors of negative mental impacts of sports injuries come from a combination of physiological and psychological components. More typical and nonmodifiable factors include aspects of development such as puberty. Coupled with psychological factors such as one's perspective of their body image along with social support, as well as unforeseen environmental factors such as the COVID-19 epidemic, these risk factors can further impact a young athlete's mental well-being and recovery process.

Age: Physiological Versus Psychological

Adolescence is a crucial period of development for everyone but even more so for athletes. Finding the balance between physiological and psychological risks for such a vulnerable age group is essential in preventing an amplified negative impact of sports injuries. High school athletes who suffered from sports injuries, specifically ACL reconstruction, reported that psychological barriers overwhelmed physical barriers, especially regarding associations of sports and injury, the fear of not making a full recovery, and social comparison to others with injury [9]. Psychological limitations and trauma were found to be greater among athletes aged 15-21 than in those 14 or younger [9]. This is likely attributed to the increasing time commitment and dedication that one has to their sport as they grow up. Even though older and more experienced athletes tend to have lower levels of anxiety and a more stable foundation mentally due to natural progress in emotional regulation and enhanced cognitive and coping skills, they are also more likely to associate with their athletic identity [8]. Thus, younger athletes are more frequently able to envision the benefits of surgical reconstruction following ACL injuries and are more cognitively flexible to cope and adapt to their injury [11].

Different developmental stages may predispose athletes to various types of mental health issues post injury [10]. Similarly, different rates of maturation signify that not all children are prepared for the same type of training at the same chronological age. Many weightlifting trainers use the process of Tanner staging, which involves distinguishing chronological and physiological age, to determine the emotional and psychological maturity to allow the athlete to continue the sport [12]. The process of growth and maturation and the timing at which children enter puberty to the adolescent stage play central roles in young athlete development and carry significant implications for athletic identity [13]. For example, early-maturing girls may possess an advantage in sports that demand greater physical strength, but they may also struggle in sports requiring endurance, agility, or aesthetic qualities. Meanwhile, in late-maturing children, the growth spurt that aligns with puberty may coincide with crucial points in their athletic careers, which may disturb their progress and selection to elite pathways [13]. In summary, the distinction between age and psychological maturity is of great importance in the resulting development during puberty.

Gender

Another risk factor that can be extremely influential in the mental impacts of sports injury is gender. Generally, female adolescents experience depression at much higher rates than male adolescents, at 23% and 8.8%, respectively [11]. This carries into sports, as female patients were seen to score lower on psychological readiness and exhibit negative outlooks on their injuries than males [14]. Thus, it has been found that female athletes are less likely to return to sports, as they often lack both intrinsic and extrinsic support from family and peers [10]. Additionally, females typically go through puberty at an earlier age. Due to these hormonal changes and the lack of cognitive development at an early age, female athletes involved in sports with an aesthetic component such as gymnastics and dance may struggle with insecurity and anxieties regarding their changing body composition [5]. This exposes them to a greater risk of adopting maladaptive behaviors such as eating disorders [5]. It is therefore essential to consider the different effects that an injury has on females and males as they endure the hormonal changes associated with puberty.

COVID-19

During the COVID-19 pandemic, many adolescent athletes in the United States were found to have increased depression and anxiety, lower levels of physical activity, and decreased quality of life scores [8]. Athletes in higher grade levels fared worse than their younger peers, likely due to the implications of college recruitment and scholarships on the horizon, all brought to a halt by an unforeseen event. Initially, it was seen that athletes who played individual sports were correlated with a greater risk for depression and anxiety. However, during the pandemic, this finding was countered as team sport athletes showed worsening depression, anxiety, and quality of life compared to those who had played individual sports [8]. This is also credited to the sudden social isolation from one's community. This recent complication in our world exposed yet another difficulty for athletes and their mental health.

Training Intensity

For all sports, older athletes and those who have committed to the sport for a longer amount of time inevitably result in increased training intensity. This vigor and dedication to the sport, which may also be derived from early specialization, may directly affect physical and psychological health by altering sleep duration and quality [15]. Adolescent athletes who sleep less than eight hours per night on average have been found to be almost twice as likely to sustain a sports-related injury [15]. The commitment to sport at the expense of other activities, along with the increasingly demanding expectations, decreased sense of personal control, and abundance of negative feedback, will lead to implications of greater mental health issues following injury [15]. The great level of attachment and interdependence that athletes feel in relation to their sport will only further the negative psychological effects following injury.

Other Risk Factors

Other risk factors may come from a variety of backgrounds. There may be negative psychological issues that develop during training and competition due to poor coaching methods and a lack of developed "sports culture" in support from family and peers [12]. The pressures to perform in various aspects, such as sports and school, are often overwhelming to many athletes and may also lead to the fear of rejection or criticism from others, implying heightened anxiety with little time for rest or recovery [5]. Furthermore, injuries that result from an intent to harm or are associated with rule violations, such as a foul in a hockey game, have been associated with more significant psychological disturbances in the injured athlete [11]. Meanwhile, factors such as financial situations and transportation have a significant influence on pediatric athletes, who are limited in their athletic participation by their academic and parental availability [13]. The combination of several risk factors on the occurrence of injuries and then the subsequent recovery process can lead to more severe psychological impacts on the adolescent athlete.

Relationships

Psychology and Physiology

One's physiological and psychological development are inherently related. In adolescence, young athletes are in the beginning stages of practicing self-evaluation and learning to integrate feedback, leaving them vulnerable to perceiving criticism or constructive feedback with the perception of being inherently flawed [5]. Thus, it is imperative that the athlete's support system is aware of how their words may be interpreted by the athlete and acts accordingly. Physiologically, removal from sport may lead to neurochemical changes that may affect mood, and the abrupt cessation of routine physical activity may precipitate a depressive episode [6]. Additionally, the growth period of adolescence has been associated with diminishing relative muscle strength, resulting in decreased coordination and neuromuscular control [16]. Together, these physiological implications of sports injury in the brain may cause a neurochemical imbalance and neuroinflammatory processes throughout the body [6]. Many athletes who show fear of reinjury during rehabilitation and beyond show elevated brain activity in the hippocampus and amygdala during motor imagery tasks [16]. This finding suggests that the movement of the injured area evokes unpleasant memories associated with the initial injury and kinesiophobia, which contributes to the reluctance of athletes to return to sport. In summary, the association between the brain and outward psychological expressions is intertwined with a sense of identity for the athlete and long-term mental security.

Athlete Identity

One's athletic identity is a measure of the degree to which one identifies with their sport. Higher levels of athletic identity are often associated with improved athletic performance, enhanced commitment, and a higher level of enjoyment of the sport [8]. However, this also means that athletes with a high athletic identity are more prone to overtraining, continued play while injured, difficulty coping with injury or the discontinuation of sports, and high-risk behaviors such as eating disorders or using performance-enhancing substances [8]. This exclusive athlete identity may also interfere with other aspects of life, as the desire to compete may override pain, fatigue, or medical advice and prompt detrimental effects of sports injuries on mental health [13]. Although sport participation may be a source of confidence and self-esteem and a mechanism for coping with life stressors, the continued drive to continue sport despite physical limitations overwhelms the rational aspect of maintaining both physical and psychological health.

Specialized Sports

Sports specialization requires an extreme commitment to one activity for extended periods of time, incorporating the sport into one's lifestyle. Despite the decreasing rates of participation in sports, the average number of hours per week is increasing in youths who do engage in sports [8]. This is credited to the increasing standard expectation of early sports specialization. Although this may be a way to increase elite performance, it can also lead to more severe psychological impacts after injury. Specialization and competitiveness can lead to injury, overtraining, and increased psychological stress [1]. This can also contribute to a decrease in the enjoyment of the sport, intense pressures to perform, and a lack of control or decision-making power, all of which are commonly cited reasons for withdrawal from sports in young athletes [5]. Elite athletes are more vulnerable to injury and burnout due to the nature of the intensity of sports and are reported to have a slower psychosocial recovery than recreational athletes [4]. Similarly, symptoms of depression are more prominent in elite athletes [4]. There are a multitude of cases of athletes who experienced early burnout and withdrawal from sports due to injury and psychological stress. For example, rhythmic gymnasts who typically train from ages four to 16 rated their health as lower and experienced less enjoyment with their sport [7]. Junior tennis players who burned out earlier were not as involved in their training, were exposed to higher levels of parental criticism and expectations, and had lower levels of motivation [7]. Finally, elite Russian swimmers cited difficult training episodes and psychological fatigue as their main reasons for dropping out of the sport [7]. These examples only emphasize the psychological difficulties over time for adolescents as they dedicate such an extreme amount of time to one sport.

School

Many adolescent athletes are pressured to do well in both their respective sports and academics. However, following injuries, many adolescents noted a decrease in grades, difficulty following classes, and an overall increase in frustration with their schoolwork [17]. Especially in sports-related concussions, the return to learning in the school environment prior to the full recovery of neurocognitive deficits will likely contribute to further adversity for the student, including social isolation, decline in academic function, and prolonged recovery time [17]. Along with the pressure that adolescents already face, the removal from a central point of their identities will inevitably result in psychological stress for the student.

Interventions

Self/Individually

Despite research that suggests that social and psychological aspects of recovery should be part of the rehabilitative process, many recovery plans only involve physiological treatments, and the athletes are thus left to establish their own coping strategies following injury [9]. There are several coping strategies that athletes can use to help mitigate psychological disturbances and improve their outlook on recovery, including self-distraction, venting, the use of emotional or instrumental support, positive reframing, and acceptance [11]. Positive reframing, the process of thinking about a negative situation with a more optimistic lens, has been shown to be the only coping strategy associated with increased rates of return to sports, decreased levels of kinesiophobia, and improved postoperative satisfaction in athletes under 20 years old who are recovering from sports-related knee injuries [11]. This positive outlook on a widely perceived bleak situation improves the mindset of athletes overall.

Support System Interventions

Although individual interventions are important for recovery, it is suggested that psychological factors during recovery encompass various types of support from parents, coaches, teammates, physical therapists, and surgeons as well [10]. It is essential that the correct type of support is given. For example, overly sympathetic support from others or insufficient attention from physical therapists or surgeons, especially in relation to a patient's goals, may negatively impact the patient's recovery experience and outcome [10]. Providers should keep gender-specific fears and motivations in mind when addressing the patient, using target-specific cognitive behavioral, motivational, and physical therapy techniques to promote the well-being of injured athletes [11]. For example, females typically focus on staying in shape, so it is advised that their emotions be monitored and social support be used more frequently, while for males, it is recommended that positive reinforcement be used to overcome their frustration with the physical limitations of injuries [11]. The desire to return to sport and self-motivation ultimately come from maintaining a positive self-concept and having the intrinsic drive to continue with your passion [14].

Learning about the recovery process as an injured athlete and interactions with the medical system may also affect the return to play in a positive manner [10]. Socialization in one's rehabilitation setting and promoting support and continued communication with the sport can help athletes build a social support system [10]. Because injuries can be so isolating for adolescent athletes whose social networks revolve around their sport, finding ways for athletes to continue participating in practice and engaging in team spirit will further encourage them to return to sport [10].

An essential aspect of treating the mental health needs of athletes is to be aware and educated as providers to foster conversations with the patient, their parents, coaches, and other sources of support across disciplines and specialties [8]. Therefore, interventions should include not only efforts involving the athlete and directly related associations but also club and league administrators and sports governing bodies in order to facilitate a successful return to sport [8]. The stigma that is often associated with needing a mental health service and confusion while navigating mental health services contributes to the lack of follow-up in athletes who are suffering from mental health consequences after injury [18]. This often leads athletes to feel additional pressure from themselves, coaches, or even media and fans to suppress their symptoms [6]. Specific individualized plans to return to sport, created by the combined efforts of parents, pediatricians, teachers, social workers, and coaches, are most likely to accelerate the recovery process while ensuring a safe return [17]. Ultimately, a combination of psychological treatments and physiotherapy is more effective than physiotherapy alone in improving psychological outcomes to encourage the athlete to return to sport in optimal condition.

Discussion

The main findings of this systematic review cover the main psychological impacts of adolescent athletes suffering from an injury in varying levels of sports. Although many physicians focus on the physical comeback of athletes, they often fail to examine the mental implications such isolation brings. The mechanism of injury, whether it be from burnout, overtraining, or pure accident, each implies different psychological impacts on young athletes. Even if short-term consequences are dealt with in rehabilitation, monitoring the patient's mentality for years following the injury is essential to ensure the development of identity as the athlete grows into adulthood, especially in puberty. Nonmodifiable risk factors, such as age and gender, are prominent aspects to consider when evaluating the psychological extent of one's injury. However, other modifiable risk factors, such as training intensity and financial situations, may also impose considerable effects on the young athlete's development after injury. Along with playing sports in the adolescent age comes the decision to dedicate oneself to the sport in the process of specialization, in which the athlete likely will encompass a separate identity to associate oneself with their sport. This, along with the additional burdens of academic success, often leads to additional stressors that can negatively affect the mental health of athletes. Not only can adolescents experience diagnosable illnesses such as depression or PTSD, but also the psychosocial effects of the sudden separation from a part of their athletic identity can include feelings of fear, doubt, and burnout. The return to sport is also often a difficult journey. It is imperative that athletes find reliable support systems to guide them through the adversities of navigating the path back to their respective sports, whether it be individual coping mechanisms or psychological treatments. The combination of physiotherapy and psychotherapy offers the optimal treatment for athletes to feel psychologically secure and ready to continue their athletic careers.

Despite the seemingly increasing interest and commitment to sports at an early age, the true participation rate of children in sports has decreased dramatically, citing financial burdens and parental distress as the main factors in this trend. However, early sports specialization is becoming more common, implying that fewer young athletes are participating in sports and a larger number of adolescents are undergoing the increasingly rigorous training programs that come with the dedication necessary to succeed. Some surprisingly influential aspects of recovery in the psychosocial aspect of adolescent athletes come from the support of parents, coaches, and other trustworthy people around the athlete. The return to sport is heavily dependent on social considerations and acceptance back into the sporting community. This includes parents who are willing to continue supporting their child even after an injury, as well as coaches and peers, with whom the athlete will seek the most acceptance when they return to find their place among the people around them. Additionally, the prominence of school on the neurocognitive development of injured athletes is immense. The building pressures from a myriad of factors in one's social world inevitably result in psychological stress. Despite the commonly held belief that psychological distress correlates with diseases such as depression and anxiety, the vast majority of injured athletes suffer more from non-diagnosable illnesses, such as a loss of confidence and a growing sense of doubt.

The strengths of this systematic review include the consideration of numerous papers covering several different types of injuries. By employing a generous search strategy, we were able to produce over 130 papers at the start of our search, which provided us with more than enough information to review. We ultimately sorted out the most impactful and influential papers, with information most relevant to our focus questions. There is surprisingly little information currently regarding the effects of injuries on the risk of developing negative psychological disorders, particularly in adolescents. This break in existing information despite the millions of athletes who participate in sports and suffer injuries every year fueled this systematic review to delve deeper into the lasting mental implications of such injuries. While past research outlined the associations between injury and mental health disorders, the goal of this paper was to promote concrete interventional trials to further progress in assisting injured athletes in a safe and efficient return to sport. Additionally, a major discrepancy arose when comparing our findings to previous ones based on the age groups. While some similarities may exist in psychological responses to injuries in adolescents and adults, the development stage of adolescence poses unique challenges that this study highlights as a perspective that has not been emphasized in past literature. Moreover, this review highlights the role of social support in aiding athletes' return to sport. Consistent with existing studies, our findings stress the immense influence that parents and peers have in decreasing the negative psychological effects of injuries. However, a gap was found in the context of adolescent sports injuries. Thus, future research should explore specific mechanisms of social support roles in relation to the young athlete. This would help to maximize the support provided by parents, coaches, peers, policymakers, and healthcare providers as they have access to information regarding how to take immediate and focused actions to enhance psychological improvement following injury.

There are also several limitations to this review. First, there was a lack of primary sources when conducting research, which can lead to the susceptibility of bias. This bias may also come from our search strategy in the PubMed database. Narrowing down the results to the last 15 years and only English-language studies may have excluded relevant studies published in other languages or outside the time frame. Although we made efforts to include a diverse range of studies, the generalizability of the findings may be another concern. Cultural differences, socioeconomic backgrounds, and different healthcare systems worldwide may have not been addressed. This topic is also rarely studied, and this lack of dedicated research poses another limitation of gaps in the research. One such gap is overlooking external psychological challenges that may affect an athlete's well-being. Additionally, when conducting research on an adolescent population, there are several ethical concerns to be considered. Maintaining confidentiality is essential, especially when discussing sensitive topics such as psychological impacts on such a young population. Researchers must ensure that all information is kept confidential. It is also paramount that informed consent is obtained, from both the adolescent athletes and their guardians if the participant is under 18. All participant information should be protected, with respect for their autonomy and privacy. Given the delicate nature of the topic, minimizing harm should be a priority for researchers and the study, being mindful of the language used and having access to psychological support at any time. Ultimately, this systematic review compiles information to convey the intricate connection between the physical and psychological aspects of adolescents and the social, emotional, financial, and other burdens athletes carry as they recover from injuries and return to their sport. In the future, we hope that this review can be used to guide true tests on ensuring the mental health of young athletes.

## Conclusions

This systematic review discusses the psychological implications of adolescent injury derived from sports. It was found that assessing both physiological and psychological aspects of the athlete's well-being is essential to making a full recovery to facilitate return to sport. The personal connections an athlete in the developmental stage feels as they go through puberty are imperative to nurture their identity, and a healthy relationship is, therefore, necessary to support an adolescent's growth. The influence of external factors such as school and environmental concerns such as COVID-19 are also significant components that can influence the psychological impacts of athletes, especially those suffering from injuries. Ultimately, physical and psychological elements are interconnected, and it is paramount that athletes and their support systems realize the significance of both aspects of recovery. Future research should focus on interventional research to promote all aspects of psychological, social, and emotional well-being in injured adolescent athletes.

## References

[1] Malm C, Jakobsson J, Isaksson A (2019). Physical activity and sports-real health benefits: a review with insight into the public health of Sweden. Sports (Basel).

[2] Shanmugam C, Maffulli N (2008). Sports injuries in children. Br Med Bull.

[3] Pfirrmann D, Herbst M, Ingelfinger P, Simon P, Tug S (2016). Analysis of injury incidences in male professional adult and elite youth soccer players: a systematic review. J Athl Train.

[4] Piussi R, Berghdal T, Sundemo D (2022). Self-reported symptoms of depression and anxiety after ACL injury: a systematic review. Orthop J Sports Med.

[5] Daley MM, Shoop J, Christino MA (2023). Mental health in the specialized athlete. Curr Rev Musculoskelet Med.

[6] Jeckell AS, Fontana RS (2021). Psychosocial aspects of sport-related concussion in youth. Psychiatr Clin North Am.

[7] Jayanthi N, Pinkham C, Dugas L, Patrick B, Labella C (2013). Sports specialization in young athletes: evidence-based recommendations. Sports Health.

[8] Daley MM, Reardon CL (2024). Mental health in the youth athlete. Clin Sports Med.

[9] Haraldsdottir K, Watson AM (2021). Psychosocial impacts of sports-related injuries in adolescent athletes. Curr Sports Med Rep.

[10] Park AL, Furie K, Wong SE (2023). Stronger athlete identity is a risk factor for more severe depressive symptoms after musculoskeletal injury in pediatric athletes: a systematic review. Curr Rev Musculoskelet Med.

[11] Patankar AG, Christino MA, Milewski MD (2022). Psychological aspects of adolescent knee injuries. Clin Sports Med.

[12] Pierce KC, Hornsby WG, Stone MH (2022). Weightlifting for children and adolescents: a narrative review. Sports Health.

[13] McKay CD, Cumming SP, Blake T (2019). Youth sport: friend or foe?. Best Pract Res Clin Rheumatol.

[14] Vutescu ES, Orman S, Garcia-Lopez E, Lau J, Gage A, Cruz AI Jr (2021). Psychological and social components of recovery following anterior cruciate ligament reconstruction in young athletes: a narrative review. Int J Environ Res Public Health.

[15] Quinn BJ, Scott C, Stracciolini A (2021). Special considerations for growing dancers. Phys Med Rehabil Clin N Am.

[16] Brindisino F, Garzonio F, DI Giacomo G, Pellegrino R, Olds M, Ristori D (2023). Depression, fear of re-injury and kinesiophobia resulted in worse pain, quality of life, function and level of return to sport in patients with shoulder instability: a systematic review. J Sports Med Phys Fitness.

[17] Neelakantan M, Ryali B, Cabral MD, Harris A, McCarroll J, Patel DR (2020). Academic performance following sport-related concussions in children and adolescents: a scoping review. Int J Environ Res Public Health.

[18] Apple RW, Stran BM, Tross B (2020). Psychologists’ role in concussion assessments for children and adolescents in pediatric practice. Int J Environ Res Public Health.

